# Bioinformatics analysis and experimental studies reveal KPNA2 as a novel biomarker of hepatocellular carcinoma progression and telomere maintenance

**DOI:** 10.1186/s40001-025-02866-z

**Published:** 2025-07-16

**Authors:** Ke Ding, Lei Liu, Wang Yong, Beicheng Sun, Wenjie Zhang

**Affiliations:** 1 Department of Hepatobiliary Surgery, Nanjing Drum Tower Hospital Clinical College of Nanjing Medical University, Nanjing, 210008 Jiangsu China; 2 Department of Hepatobiliary Surgery, The First Affiliated Hospital of Anhui Medical University, Hefei, 230022 Anhui China; 3 MOE Innovation Center for Basic Research in Tumor Immunotherapy, Hefei, 230022 China; 4 Anhui Province Key Laboratory of Tumor Immune Microenvironment and Immunotherapy, Hefei, 230022 China

**Keywords:** Hepatocellular carcinoma, Prognosis, Telomere maintenance, KPNA2, Bioinformatic analysis

## Abstract

**Background:**

Telomere maintenance mechanisms (TMMs) play a distinct role in the initiation and progression of hepatocellular carcinoma (HCC). However, the prognostic relevance of telomere maintenance (TM)-related genes in HCC remains largely unclear.

**Methods:**

We integrated expression profiles of TM-related genes and corresponding clinicopathological data from public databases. Univariate analyses were performed to identify prognostic genes, and Cytoscape software was used to validate hub genes within the TM-related network. A novel prognostic signature was then constructed using the LASSO Cox regression algorithm. Finally, in vitro experiments were conducted to explore the functional roles of the key hub gene KPNA2 in telomere maintenance, tumor growth, and metastasis in HCC.

**Results:**

In this study, we identified 224 differentially expressed TM-related genes for the first time. Functional enrichment and pathway analyses revealed that these genes were highly involved in telomere-associated pathways, including cell proliferation and cellular senescence. Protein–protein interaction (PPI) analysis identified eight hub TM-related genes (RNASEH2A, KPNA2, AURKB, FOXM1, MKI67, RAD54L, PLK1, and KIF4A), all of which were positively correlated with telomere maintenance. Furthermore, a novel TM-related prognostic signature comprising seven genes (KPNA2, CACNA1B, IRAK1, CDCA8, RGMA, ETS2, and GNE) was developed using the LASSO Cox model. Notably, KPNA2 was identified as both a TM-related hub gene and a component of the prognostic signature. KPNA2 was found to be significantly upregulated in HCC and associated with poor clinical outcomes. Functional assays revealed that KPNA2 knockdown suppressed telomerase activity, inhibited tumor cell proliferation and metastasis, whereas its overexpression produced the opposite effects. Telomerase inhibition partially alleviated the inhibitory effect of KPNA2 overexpression on cell proliferation and migration.

**Conclusions:**

This study identified eight TM-related hub genes with prognostic significance in HCC and established a novel TM-related gene signature. Furthermore, we validated KPNA2 as a key regulator of telomere maintenance and tumor progression in HCC, suggesting it as a potential therapeutic target for improving clinical management of HCC.

**Supplementary Information:**

The online version contains supplementary material available at 10.1186/s40001-025-02866-z.

## Introduction

Hepatocellular carcinoma (HCC), accounting for a large portion of primary liver cancer [1, 2], is the fourth leading cause of cancer mortality worldwide, with the five-year survival rate below 20% [3, 4]. Many efforts have been put into identifying biomarkers to enhance the prognostic accuracy of HCC patients [5]. However, the complicated etiology and high degree of heterogeneity impose restrictions on prognosis prediction and treatment. Most new biomarkers have not been settled into daily practice. Thus, there is a constant search for more superior biomarkers and effective predictive models to prolong patients’ survival time with HCC. Previous studies have demonstrated that more than 80% of HCCs can reactivate telomerase, highlighting the unique role of telomere biology in the formation and development of HCC [6].

Telomeres are protective nucleoprotein complexes localized at the ends of linear chromosomes that consist of a GT-rich DNA repeat sequence (5′-TTAGGG-3′) [7]. The essential role of telomeres is to protect the ends of eukaryotic chromosomes, inhibit the DNA damage response, and maintain genomic stability and integrity [8]. Somatic cell proliferation can cause telomere shortening, which ultimately leads to senescence or apoptosis [9]. Therefore, cancer cells require telomere maintenance mechanisms for unlimited proliferative potential, which include telomerase-mediated maintenance, alternative lengthening of telomeres (ALT), and non-defined telomere maintenance mechanism (NDTMM) [10–12]. The main component of telomerase is telomerase reverse transcriptase (TERT) [13]. Telomerase synthesizes telomeric DNA repeats using its RNA template and prevents chromosome shortening [14]. ALT is a TMM that involves homologous recombination or homology-directed repair mechanism [15]. TMM plays a crucial role in the initiation and progression of HCC and could be a potential prognostic biomarker for HCC patients. However, the previous studies related to TMM in HCC mainly focused on the TERT promoter mutations. This study revealed the prognostic impact of telomere maintenance (TM) related genes in HCC, as well as their potential characteristics in HCC.

Our study identified eight TM-related hub genes with prognostic significance in hepatocellular carcinoma (HCC) and established a novel TM-related gene prognostic signature. Notably, KPNA2 was identified as both a TM-related hub gene and a component of the prognostic model. KPNA2 was found to be significantly upregulated in HCC and associated with poor clinical outcomes. Functional analyses demonstrated that KPNA2 knockdown inhibited telomerase activity, tumor cell proliferation, and metastasis, whereas its overexpress.

## Materials and methods

### Data retrieval and preprocessing

Bulk RNA-seq data and the corresponding clinical data were obtained from TCGA database (https://portal.gdc.cancer.gov/repository) and ICGC database (https://dcc.icgc.org/projects/LIRI-JP). TCGA dataset contains HCC data of 374 primary tumor samples and 50 normal tissue samples. ICGC dataset contains data of 240 tumor samples and 202 normal samples. The raw read counts data were used in the study.

We obtained the TM-related gene list from the TelNet database [16] (http://www.cancertelsys.org/telnet/). The list contains a total of 2093 human TM-related genes. Then, we filtered the two datasets with the TM-related gene list to obtain 1919 TM-related genes for the analysis.

### Differential gene analysis

We used the “Deseq2” R package to identify the differentially expressed TM-related genes between tumor samples and normal samples in both TCGA and ICGC datasets, respectively [17]. Genes with log2|FC|≥ 1 and padj ≤ 0.01 were identified as differential expressions. Differentially expressed TM-related genes from the two datasets were then extracted and intersected together.

### Functional analyses with differentially expressed TM-related genes

In order to explore significant molecular functions, biological processes, cellular components, and signaling pathways of these differentially expressed TM-related genes, gene ontology (GO) and Kyoto Encyclopedia of Gene and Genome (KEGG) pathway analyses were conducted using the “clusterProfiler” R package [18].

### Identification of hub TM-related genes

To guarantee the quality of this study, patients with OS < 30 days and lack of clinical information were excluded from the TCGA dataset. Finally, 318 HCC patients were included in further analysis. We employed a univariate Cox regression model on each differentially expressed TM gene. The survival analysis was implemented by “survival” R package. The genes are considered significantly related to the patients’ overall survival if the p-value of the Cox model is less than 0.01.

To further select the core regulatory genes among these prognostic DEGs, a protein–protein interaction (PPI) network based on differentially expressed TM-related genes related to the patients’OS was constructed using the STRING v11.0 database (https://stringdb.org/) [19]. The minimum confidence score was set at 0.400, and the disconnected nodes were hidden in the network. The nodes represent the identified core genes, and the edges indicate interactions between them. The size of each node is proportional to its degree of connectivity within the network.

Cytoscape software (version 3.7.2) was used to presents the results from STRING [20]. Twelve topological analysis algorithms from the CytoHubba app plugin of Cytoscape was used to verify hub genes within the entire network, including Degree, Maximal Clique Centrality (MCC), Density of Maximum Neighborhood Component (DMNC), Maximum Neighborhood Component (MNC), Edge Percolated Component (EPC), BottleNeck, EcCentricity, Closeness, Radiality, Betweenness, Stress, and ClusteringCoefficient [21]. “UpSetR” R package was used to intersect and visualize the outcome [22].

### Construction and validation of the TM-related prognostic model

TCGA dataset was used to construct the prognostic model. Normalized read counts were extracted from the Deseq2 package and z-score transformation is implemented. Survival related TM-related genes were used as the variables for the multiple Cox regression model. To minimize the risk of overfitting, we first implemented the stepwise regression model and then applied the least absolute shrinkage and selection operator (LASSO) Cox regression analysis with the"glmnet"R package in order to determine the final regression model [23]. The risk scores were calculated according to the following formula:

$${\varvec{score}}={\varvec{exp}}\{{\varvec{sum}}({\varvec{expressional}}\boldsymbol{ }\,{\varvec{level}}\,\boldsymbol{ }{\varvec{of}}\boldsymbol{ }{\varvec{each}}\boldsymbol{ }{\varvec{gene}}\boldsymbol{ }\times \boldsymbol{ }{\varvec{corresponding}}\boldsymbol{ }{\varvec{coefficient}})\}$$

The median value of the risk score was set as the cut-off point to stratify patients into high-risk and low-risk groups. Kaplan–Meier survival analysis was constructed to evaluate the survival differences of patients between high-risk and low-risk group. The"survivalROC"R package was used to conduct the time-dependent receiver operating characteristic (ROC) curves to evaluate the predictive performance of the prognostic model. To verify the results, we validated the prognostic model in an independent dataset (ICGC).

### Establishment and validation of a nomogram model

Univariate and multivariate Cox regression analyses were carried out on both the TCGA training dataset and the ICGC validation dataset to assess whether the prognostic signature can become an independent prognostic factor.

A predictive nomogram with independent prognostic factors was established using the"rms"R package to evaluate the 1-, 3-, and 5-year OS in HCC patients [24]. The Harrell’s concordance index (C-index) and the calibration plot were used to evaluate the discrimination and consistency of the nomogram.

### Cell lines and culture

Human hepatocellular carcinoma (HCC) cell lines—Hep3B was obtained from the Shanghai Institute for Biological Sciences (China). All cell lines were cultured in Dulbecco’s Modified Eagle Medium (DMEM) supplemented with 10% fetal bovine serum (FBS), 100 U/mL penicillin, and 100 µg/mL streptomycin. Cells were maintained at 37 °C in a humidified incubator with 5% CO₂.

### Cell transfection

Lentiviral vectors encoding KPNA2, or corresponding shRNAs were transduced into HCC cells in six-well plates using 10 µg/mL polybrene, followed by a 24-h incubation. shRNA sequences are provided in Supplementary Table 1.

### Cell viability assays

Cell viability was assessed using a CCK-8 kit (Vazyme, #A311) and an EdU Cell Proliferation Kit with Alexa Fluor 488 (Beyotime, #C0075). For CCK-8 assays, 1000 cells were seeded per well in 96-well plates and incubated overnight. Following two PBS washes, 10 µL of CCK-8 reagent and 90 µL of serum-free medium were added. After 1 h of incubation at 37 °C, absorbance at 450 nm was measured using a microplate reader. For EdU assays, 3 × 10^5^ cells were seeded per well in 24-well plates and processed according to the manufacturer's instructions. Fluorescent images were captured using a fluorescence microscope.

### Transwell migration and invasion assays

Cell migration were evaluated using Transwell chambers. A total of 1 × 10^5^ cells were seeded in the upper chamber, while the lower chamber contained 600 µL of DMEM with 10% FBS. After 48 h of incubation, non-migrated cells on the upper membrane surface were removed with a cotton swab. The membranes were fixed with 4% formaldehyde for 15 min, stained with crystal violet, and imaged under a light microscope.

### Wound-healing assay

To assess cell migration, 1 × 10^5^ cells were plated into each well of an Ibidi Culture-Insert 2 Well insert placed in a 24-well plate. Once cells adhered, the insert was removed using sterile tweezers. After two PBS washes, DMEM containing 1% FBS was added. Wound closure was monitored and imaged using an Olympus optical microscope in conjunction with the MShot Image Analysis System.

### RNA isolation and quantitative real-time PCR

Total RNA from liver tissues and cultured cells was extracted using TRIzol reagent (Invitrogen, #15,596,018) following the manufacturer’s protocol. Reverse transcription was performed using HiScript III RT SuperMix (Vazyme, #R323-01). Quantitative PCR was conducted with ChamQ Universal SYBR qPCR Master Mix (Vazyme, #Q711-02) on an Applied Biosystems 7300 Detection System. β-actin served as the internal control. Primer sequences are listed in Supplementary Table 2.

### Western blot analysis

Proteins were extracted from cells and liver tissues using ice-cold RIPA buffer containing PMSF (Beyotime, #ST507) and phosphatase inhibitors (KeyGEN, #KGP602). Protein concentrations were determined with a BCA assay. Samples were separated by SDS-PAGE and transferred onto PVDF membranes (Bio-Rad). Membranes were blocked with 5% BSA in TBST, incubated with primary antibodies, followed by HRP-conjugated secondary antibodies. Proteins were visualized using an enhanced chemiluminescence detection kit (Vazyme, #E412-01). Antibody details are provided in Supplementary Table 3.

### Immunofluorescence and immunohistochemistry

For immunofluorescence, 4 µm-thick frozen tissue sections were fixed in 4% paraformaldehyde for 30 min. After permeabilization and blocking, sections were incubated overnight at 4 °C with primary antibodies, followed by Alexa Fluor 488-conjugated anti-rabbit secondary antibodies for 2 h at room temperature. Nuclei were stained with DAPI for 20 min. Images were acquired using a Leica TCS SP8 confocal microscope and analyzed with Leica Application Suite X software. For immunohistochemistry, paraffin-embedded sections were deparaffinized, rehydrated, and subjected to antigen retrieval. Sections were incubated overnight at 4 °C with primary antibodies, followed by biotin-conjugated secondary antibodies. Detection was performed using 3,3′-diaminobenzidine (DAB) and counterstained with hematoxylin. Antibody information is listed in Supplementary Table 3.

### Statistical analysis

Statistical analysis was implemented on R software (https://www.r-project.org/, v4.0.2) and GraphPad Prism 9 (GraphPad Inc., La Jolla, CA, USA). If not specified above, two-tailed *P*-values less than 0.05 were considered statistically significant. The association between KPNA2 expression and clinicopathological characteristics in HCC patients was evaluated using the chi-square (*χ*^2^) test. A *p*-value < 0.05 was considered statistically significant, *p < 0.05, **p < 0.01, ***p < 0.001, ****p < 0.0001.

## Results

### Identification of hub TM-related genes in HCC

**Fig. 1:**
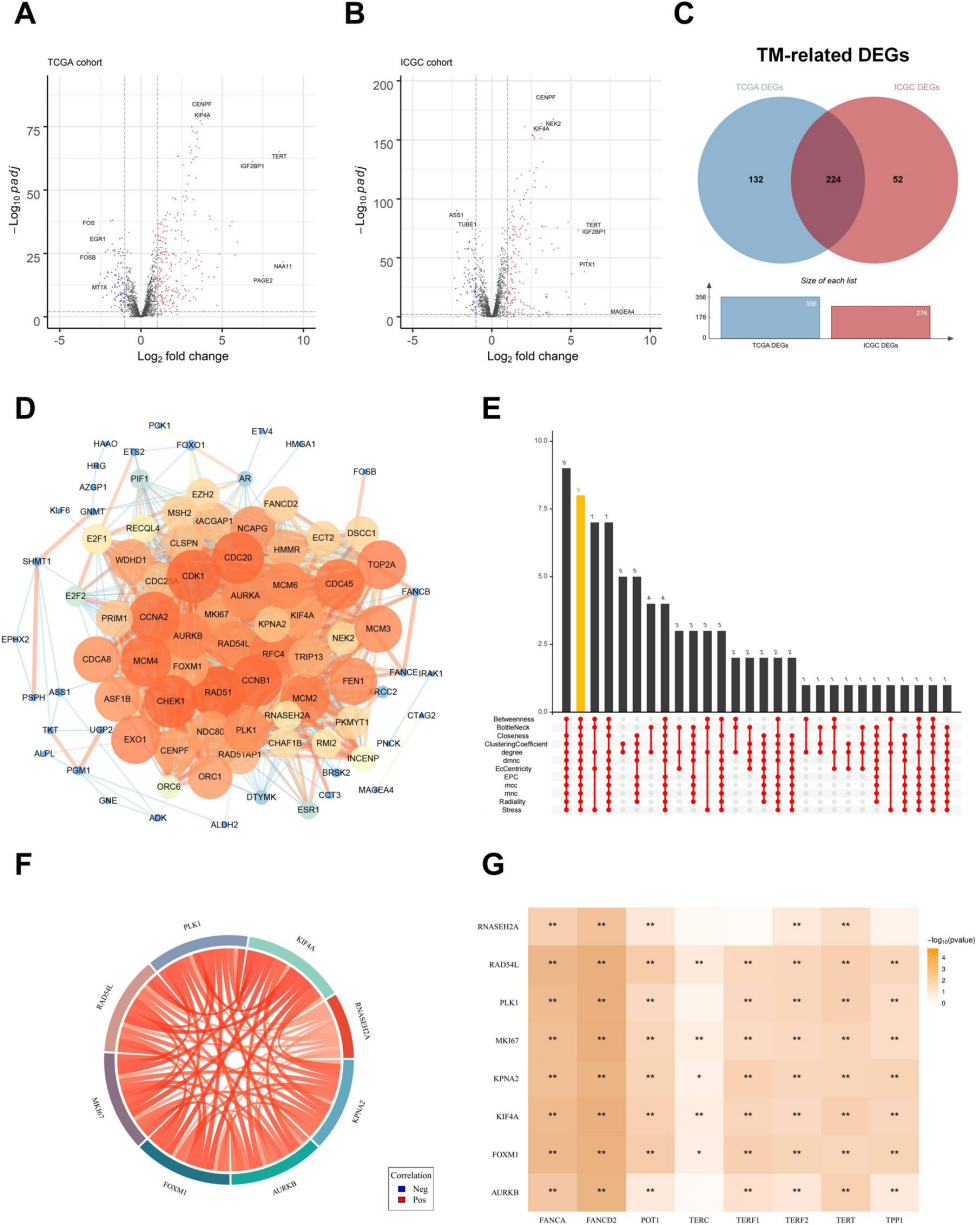
Identification of hub TM-related genes in HCC. **A**, **B** Volcano plot illustrating differentially expressed TM-related genes between HCC tumor samples and normal samples in TCGA and ICGC cohorts. **C** Venn diagram of the overlapped differentially expressed TM-related genes between two datasets. **D** The PPI network constructed through the STRING database and Cytoscape indicated the interactions among the 102 TM-related genes significantly related to OS of HCC patients. **E** Hub genes were identified by the intersection of 50 genes from 12 algorithms, including MCC, DMNC, MNC, Degree, EPC, BottleNeck, EcCentricity, Closeness, Radiality, Betweenness, Stress, and ClusteringCoefficient. **F** The relationship among eight genes. **G** The relationship between eight genes and telomerase-related genes

Due to the limited availability of normal tissue samples in the TCGA database, both the TCGA and ICGC datasets were utilized to identify differentially expressed genes (DEGs) [25]. A total of 2,093 telomere maintenance (TM)-related genes were obtained from the TelNet database. These genes were used to filter the TCGA and ICGC datasets, resulting in a final set of 1,919 TM-related genes for subsequent analyses. By comparing mRNA expression profiles between HCC and normal tissue samples in the TCGA and ICGC datasets, 356 and 276 TM-related DEGs were identified, respectively, using volcano plot analysis (log₂|FC|≥ 1, adjusted *p* ≤ 0.01) (Fig. 1A, B). The overlapping DEGs from both datasets were then identified through intersection analysis (Fig. 1C), yielding a total of 224 differentially expressed TM-related genes, including 160 upregulated and 64 downregulated genes.

To explore the biological significance of these 224 genes, Gene Ontology (GO) and Kyoto Encyclopedia of Genes and Genomes (KEGG) enrichment analyses were performed. Among biological processes (BP), DNA replication emerged as the most significantly enriched term. In the cellular component (CC) category, chromosomal region was the most prominent. For molecular functions (MF), catalytic activity, acting on DNA was the most enriched term (Supplementary Fig.  1 A). KEGG pathway analysis revealed significant enrichment in the cell cycle and cellular senescence pathways (Supplementary Fig. 1B).

To identify potential hub TM-related genes, expression data from 318 TCGA-LIHC samples and 240 ICGC-LIRI-JP samples were analyzed. Baseline characteristics of the included patients are summarized in Supplementary Table 4. Among the 224 DEGs, 102 genes were found to be significantly associated with overall survival (*p* < 0.01) based on univariate Cox regression analysis (Supplementary Table 5). A protein–protein interaction (PPI) network comprising these 102 genes (nodes) and 1211 interactions (edges) was constructed using the STRING database and visualized with Cytoscape (Fig. 1D). To further identify hub genes, 12 topological algorithms available in the CytoHubba plugin of Cytoscape were employed: MCC, DMNC, MNC, Degree, EPC, BottleNeck, EcCentricity, Closeness, Radiality, Betweenness, Stress, and Clustering Coefficient. The top 50 genes from each method were extracted, and the intersection of these sets revealed eight hub TM-related genes: *RNASEH2A, KPNA2, AURKB, FOXM1, MKI67, RAD54L, PLK1,* and *KIF4A* (Fig. 1E).

We validated the mRNA expression levels of the eight hub telomere maintenance (TM)-related genes using data from the TCGA database. All eight hub genes were significantly upregulated in hepatocellular carcinoma (HCC) tissues compared to corresponding normal liver tissues (*P* < 0.05; Supplementary Fig.  2 A). To further assess the relationship between these hub genes and telomere-associated functions, we performed gene expression correlation analyses. The results demonstrated strong positive correlations not only among the eight hub TM-related genes themselves, but also with key components of telomerase (*TERT*, *TERC*), the Shelterin complex (*TERF1*, *TERF2*, *POT1*, *TPP1*), and genes implicated in the alternative lengthening of telomeres (ALT) pathway (*FANCA*, *FANCD2*) (Fig. 1F, G). These findings were further corroborated by data from the GEPIA database, which confirmed statistically significant positive correlations between the expression levels of the eight hub TM-related genes and the broader telomere maintenance signature, including *TERT*, *TERC*, *TERF1*, *TERF2*, *POT1*, *TPP1*, *FANCA*, and *FANCD2* (Supplementary Fig. 2B). These findings strongly suggest that the identified hub genes are closely involved in the regulation and maintenance of telomere function in HCC.

### Construction and validation of the prognostic TM-related gene signature

Telomere maintenance in tumor cells is closely associated with patient prognosis. To identify TM-related genes most strongly correlated with clinical outcomes, we applied stepwise regression modeling and LASSO penalized Cox regression analysis to construct a TM-related prognostic signature. As a result, a seven-gene prognostic signature was established, and a corresponding risk score formula was developed (Supplementary Table 6, Supplementary Fig.  3 A). GSEA analysis revealed significant enrichment in the cell cycle and cellular senescence pathways in the high-risk group (Supplementary Fig.  1 C). The risk score was calculated as follows: ${\varvec{risk}}\boldsymbol{ }{\varvec{score}}=\mathbf{exp}\{\left[{\varvec{level}}\boldsymbol{ }{\varvec{of}}\boldsymbol{ }{\varvec{KPNA}}2\boldsymbol{ }{\varvec{expression}}\times \boldsymbol{ }\left(0.243777819\right)\right]+\left[{\varvec{level}}\boldsymbol{ }{\varvec{o}}{\varvec{f}}\boldsymbol{ }{\varvec{CACNAB}}\boldsymbol{ }{\varvec{expression}}\times \boldsymbol{ }\left(0.009296239\right)\right]+\left[{\varvec{level}}\boldsymbol{ }{\varvec{o}}{\varvec{f}}\boldsymbol{ }{\varvec{RGMA}}\boldsymbol{ }{\varvec{expression}}\times \boldsymbol{ }\left(-0.031361508\right)\right]+\left[{\varvec{level}}\boldsymbol{ }{\varvec{of}}\boldsymbol{ }{\varvec{I}}{\varvec{RAK}}1\boldsymbol{ }{\varvec{expression}}\times \boldsymbol{ }\left(0.012413379\right)\right]+\left[{\varvec{level}}\boldsymbol{ }{\varvec{of}}\boldsymbol{ }{\varvec{E}}{\varvec{T}}{\varvec{S}}2\boldsymbol{ }{\varvec{expression}}\times \boldsymbol{ }\left(-0.007062245\right)\right]+\left[{\varvec{level}}\boldsymbol{ }{\varvec{of}}\boldsymbol{ }{\varvec{GNE}}\boldsymbol{ }{\varvec{expression}}\times \boldsymbol{ }\left(-0.023405336\right)\right]+\left[{\varvec{level}}\boldsymbol{ }{\varvec{of}}\boldsymbol{ }{\varvec{CDCA}}8\boldsymbol{ }{\varvec{expression}}\times \boldsymbol{ }\left(0.168401787\right)\right]\}$**.**

Among these seven genes, *KPNA2*, *CACNA1B*, *IRAK1*, and *CDCA8* were negative associated with survival, while *RGMA*, *ETS2*, and *GNE* were positively prognostic markers (Supplementary Fig. 3B). Patients were stratified into high-risk and low-risk groups based on the median value of the calculated risk score. The expression patterns of the seven genes included in the prognostic signature within the TCGA cohort are illustrated in the heatmap (Supplementary Fig.  3 C). Kaplan–Meier survival analysis revealed that patients in the high-risk group had significantly shorter overall survival (OS) and higher mortality compared to those in the low-risk group (P < 0.0001; Supplementary Fig. 3D). To assess the predictive performance of the TM-related gene signature, we conducted receiver operating characteristic (ROC) curve analysis. The area under the curve (AUC) for time-dependent ROC curves was 0.794 at 1 year, 0.734 at 3 years, and 0.703 at 5 years, indicating good prognostic accuracy (Supplementary Fig. 3E). To validate the robustness of the prognostic signature, the same risk score formula was applied to the ICGC cohort. Patients in this cohort were similarly stratified into high- and low-risk groups based on the median risk score. Consistent with TCGA findings, Kaplan–Meier analysis demonstrated that high-risk patients had significantly poorer OS than those in the low-risk group (*P* < 0.0001; Supplementary Fig.  3 F, G). Furthermore, the AUCs for the ICGC cohort were 0.756 at 1 year, 0.772 at 3 years, and 0.762 at 5 years, confirming the high reliability and predictive sensitivity of the TM-related gene signature across independent datasets (Supplementary Fig. 3H).

Univariate and multivariate Cox regression analyses were performed to determine whether the TM-related prognostic signature could serve as an independent prognostic factor for overall survival (OS). In the TCGA cohort, both univariate and multivariate analyses demonstrated that the prognostic signature (Univariate: HR = 4.703, 95% CI 3.198–6.915, *p* < 0.001; Multivariate: HR = 4.563, 95% CI 2.880–7.229, *p* < 0.001) and tumor stage (Univariate: HR = 3.071, 95% CI 2.076–4.545, *p* < 0.001; Multivariate: HR = 2.299, 95% CI 1.485–3.559, *p* < 0.001) were independent prognostic indicators in HCC patients (Supplementary Fig.  4 A, B). Similarly, in the ICGC cohort, the TM-related prognostic signature remained an independent predictor of OS (Univariate: HR = 2.871, 95% CI 1.942–4.243, *p* < 0.001; Multivariate: HR = 3.818, 95% CI 2.313–6.301, *p* < 0.001) (Supplementary Fig.  4 C, D). Based on the two independent prognostic factors—tumor stage and the risk score—a telomere maintenance-related gene nomogram was constructed using the TCGA cohort to quantitatively predict individual OS probability. The concordance index (C-index) of the nomogram was 0.743 (*p* < 0.001), indicating good predictive accuracy (Supplementary Fig. 4E). Calibration plots demonstrated strong agreement between the predicted and observed survival outcomes, particularly for short-term survival, suggesting that the nomogram provides more accurate predictions for early survival outcomes than for long-term prognosis (Supplementary Fig.  4 F).

### KPNA2 is overexpressed in HCC tissues and related to telomere maintenance

**Fig. 2:**
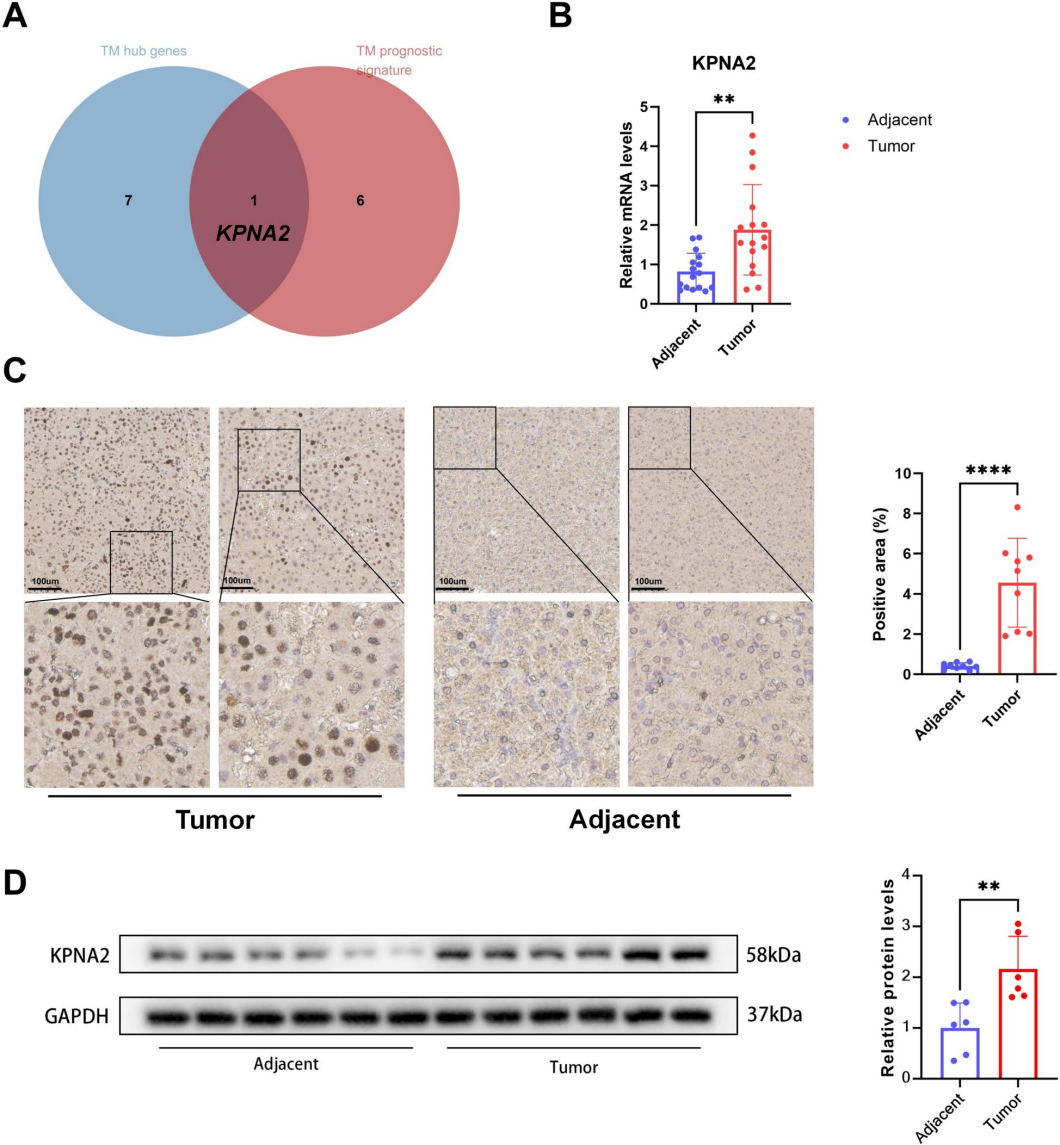
*KPNA2* is upregulated in HCC tissues. **A** Venn diagram show *KPNA2* was identified as both a TM-related hub gene and a component of the prognostic signature. **B** Related mRNA expression of *KPNA2* in HCC and adjacent peritumor tissues tested by qRT‒PCR. **C** *KPNA2* expression in HCC tissues and adjacent peritumor tissues was tested by IHC assays. **D** Related protein expression of *KPNA2* in tumor and adjacent peritumor tissues tested by Western blot

Based on the analyses of the eight hub TM-related genes and the seven-gene TM-related prognostic signature, we found that *KPNA2* was identified as both a TM-related hub gene and a component of the prognostic signature (Fig. 2A). This dual role suggests that *KPNA2* is a key gene involved not only in telomere maintenance within hepatocellular carcinoma (HCC) cells but also in significantly influencing patient prognosis. To further investigate the expression and functional role of *KPNA2* in HCC, we initially examined *KPNA2* mRNA expression in 16 pairs of tumor and adjacent peritumor tissues from HCC patients using qRT-PCR (Fig. 2B). The results showed that *KPNA2* mRNA expression was significantly elevated in tumor tissues compared to adjacent normal tissues (Fig. 2C). Subsequently, Immunohistochemistry (IHC) was conducted to investigate the protein expression of *KPNA2*. The *KPNA2* protein level was increased in tumors compared to adjacent peritumor tissues. Similarly, western blot results consistently confirmed a signifcant upregulation of *KPNA2* at the protein level in HCC tissues compared to adjacent peritumor tissues (Fig. 2D).

**Fig. 3:**
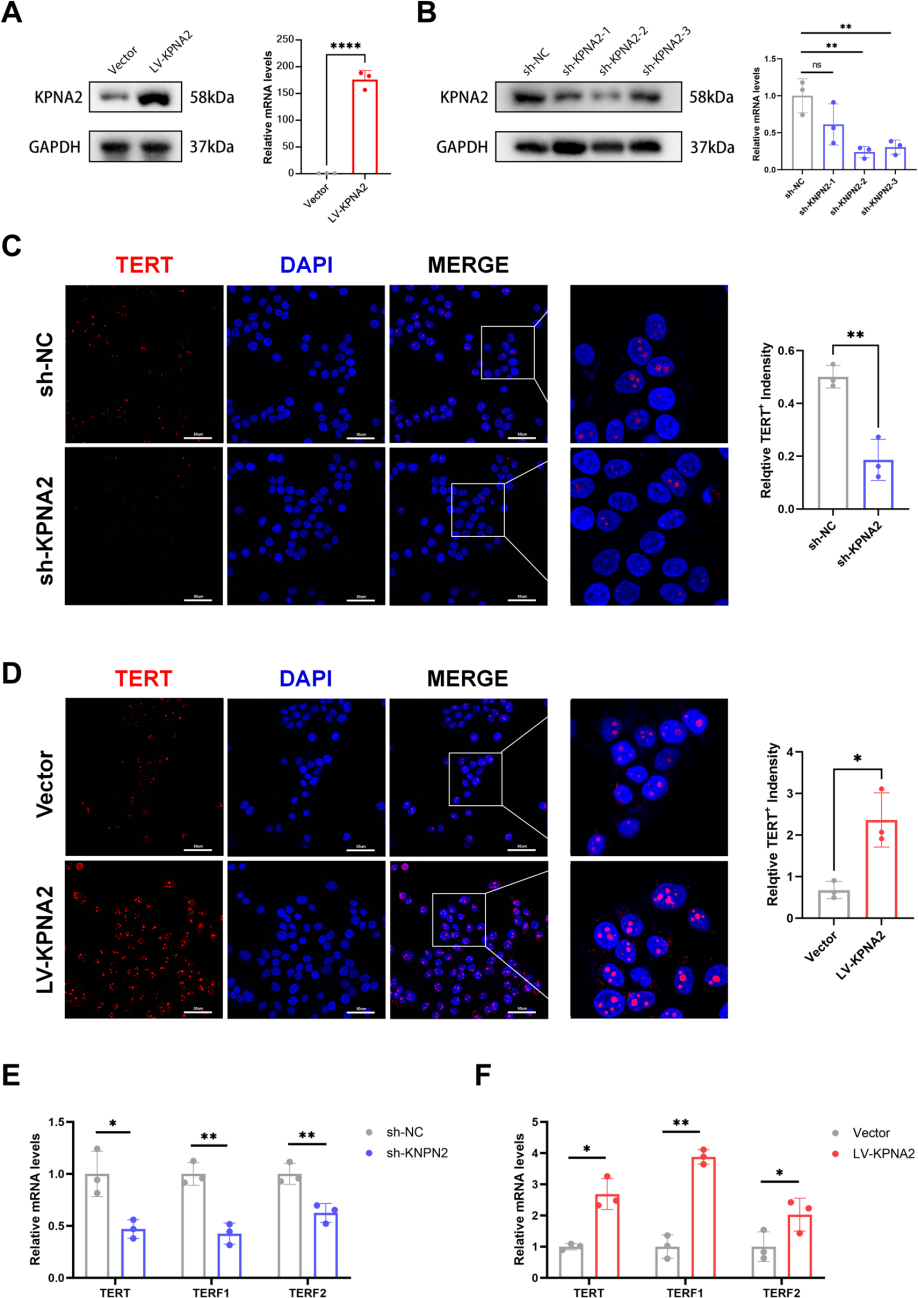
The functional role of *KPNA2* in telomere maintenance. **A**, **B** The efficacy of *KPNA2* knockdown and overexpression in Hep3B cells verifed by Western blot and qRT-PCR. **C**, **D** *TERT* expression level in *KPNA2* knockdown and overexpression cells were tested by IF assays. **E**, **F** Telomerase related genes expression level in *KPNA2* knockdown and overexpression cells were tested by qRT-PCR

To further investigate the functional role of *KPNA2* in telomere maintenance and HCC progression, we conducted *KPNA2* knockdown and overexpression experiments in Hep3B hepatocellular carcinoma cells. The efficiency of both knockdown and overexpression was confirmed by Western blot and qRT-PCR analyses (Fig. 3A, B). Immunofluorescence staining revealed that *KPNA2* knockdown suppressed the expression of telomerase (TERT) in Hep3B cells, while *KPNA2* overexpression enhanced *TERT* expression (Fig. 3C, D). Additionally, qRT-PCR analysis showed that *KPNA2* knockdown significantly downregulated the mRNA levels of TERT and two key Shelterin complex components, *TERF1* and *TERF2* (Fig. 3E). Conversely, *KPNA2* overexpression led to an upregulation of these telomere maintenance-related genes (Fig. 3F). These findings suggest that high *KPNA2* expression in HCC contributes to the maintenance of telomere function, potentially through the regulation of telomerase and Shelterin complex components.

### KPNA2 promotes HCC proliferation and metastasis via telomere maintenance

**Fig. 4:**
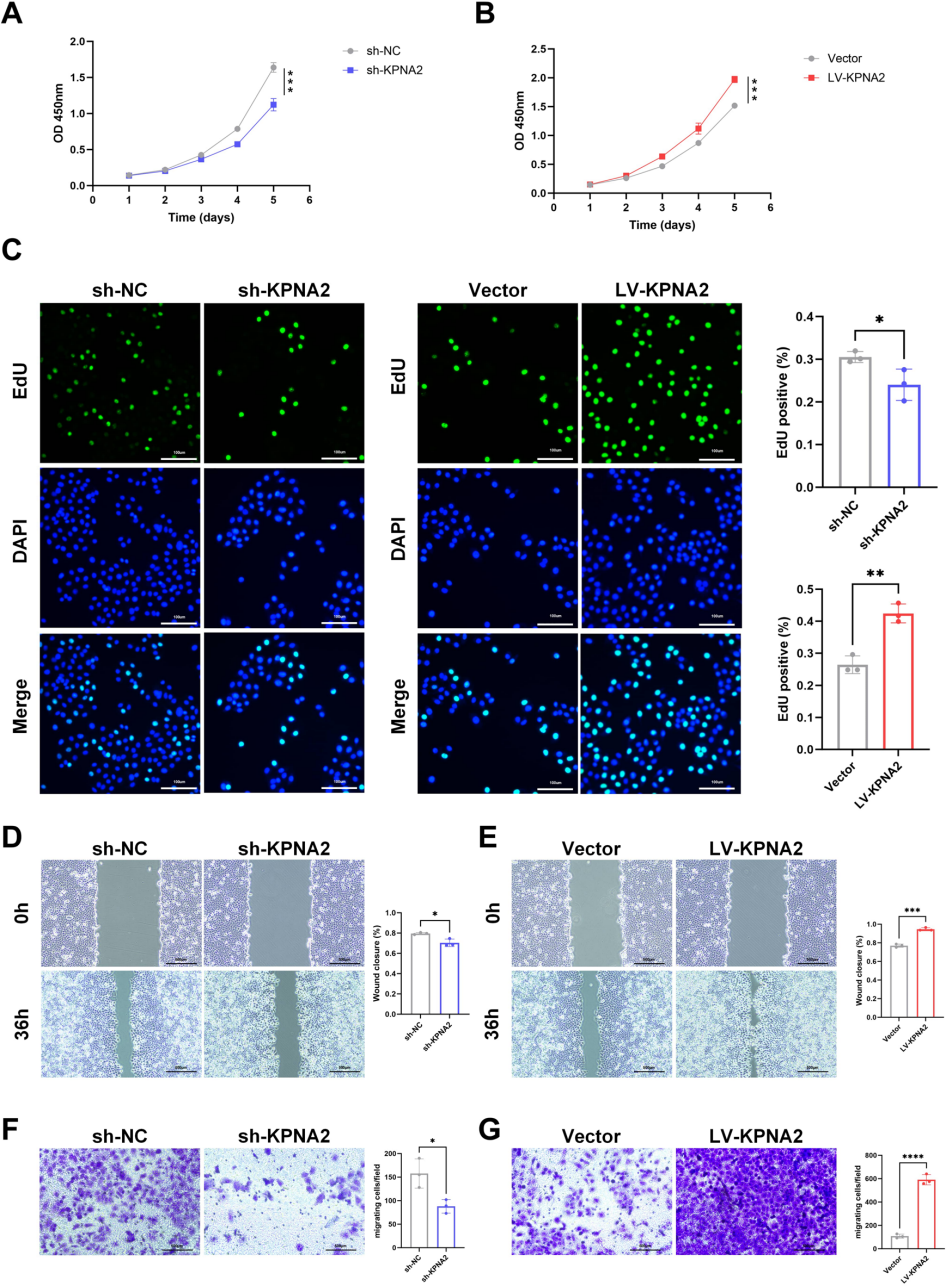
The functional role of *KPNA2* in HCC progression. **A**, **B** CCK8 assays of knockdown or overexpression cells. **C** EdU assays results of knockdown or overexpression cells. **D**, **E** Wound healing assays were performed to assess the effect of *KPNA2* knockdown or overexpression. **F**, **G** Representative image of migrated Hep3B cells after *KPNA2* knockdown or overexpression

To evaluate the role of *KPNA2* expression levels on HCC proliferation and migration, we performed CCK8 and EdU experiments. The results showed that *KPNA2* knockdown inhibited Hep3B cell proliferation, whereas *KPNA2* overexpression promoted cell proliferation (Fig. 4A–C). Transwell and wound healing assays further showed that *KPNA2* knockdown reduced the migratory capacity of Hep3B cells, while *KPNA2* overexpression enhanced cell migration (Fig. 4D–G).

**Fig. 5:**
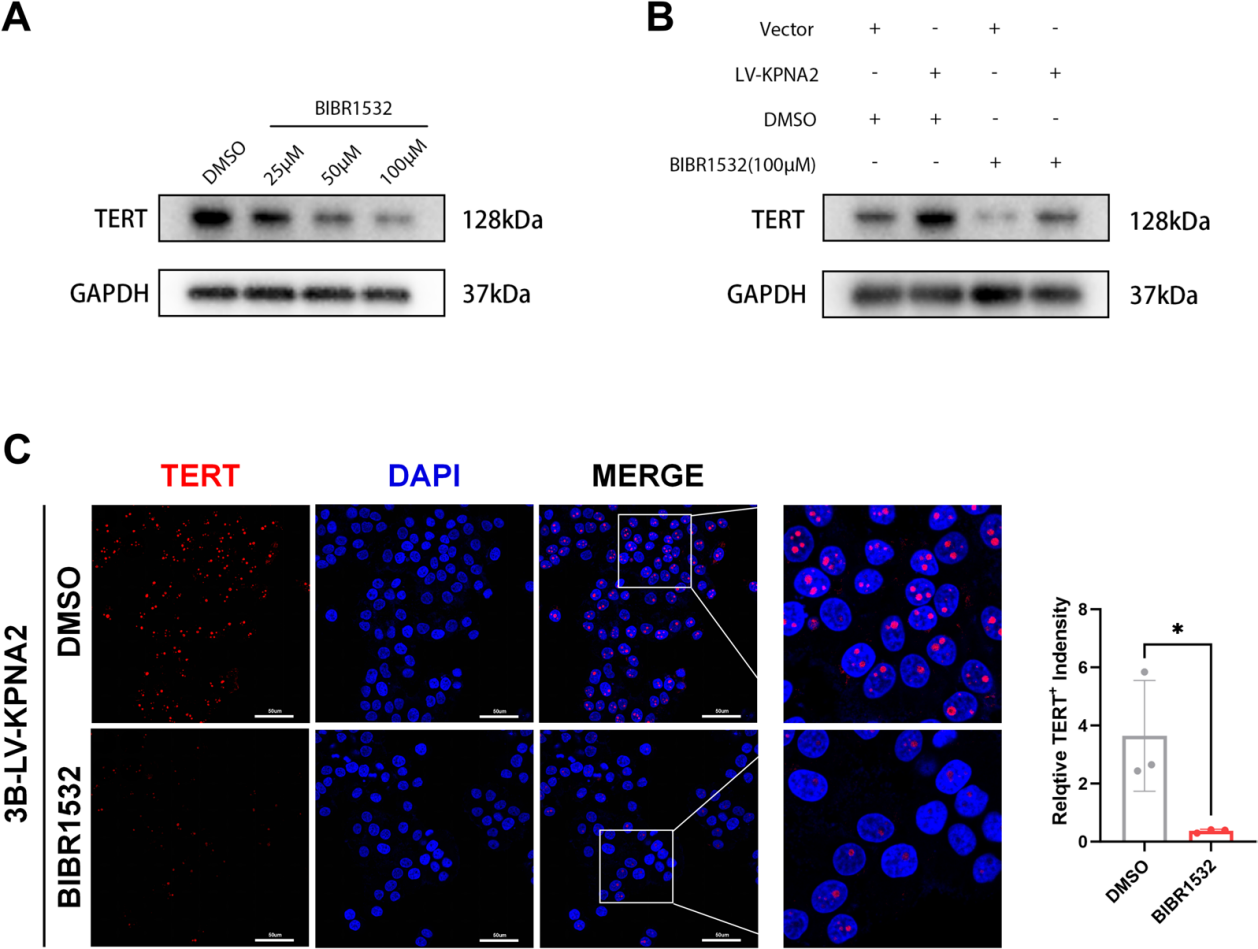
BIBR1532 downregulated TERT expressions in 3B cells. **A** BIBR1532 significantly downregulated the expression of TERT in a dose-dependent manner.** B**, **C** BIBR1532 suppressed TERT expression in cells with KPNA2 overexpression

Given the critical role of telomere maintenance in regulating tumor cell proliferation and invasion, we investigated its involvement in KPNA2-mediated HCC progression by treating HCC cells with the telomerase inhibitor BIBR1532, a well-characterized small molecule inhibitor of telomerase. The results demonstrated that BIBR1532 significantly downregulated the expression of TERT, the core catalytic subunit of telomerase, in a dose-dependent manner (Fig. 5A). Subsequently, HCC cells overexpressing KPNA2 and vector controls were treated with BIBR1532 at a concentration of 100 μM. Overexpression of KPNA2 led to an increase in TERT expression, whereas treatment with 100 μM BIBR1532 markedly suppressed TERT expression even in cells with KPNA2 overexpression (Fig. 5B, C).

**Fig. 6:**
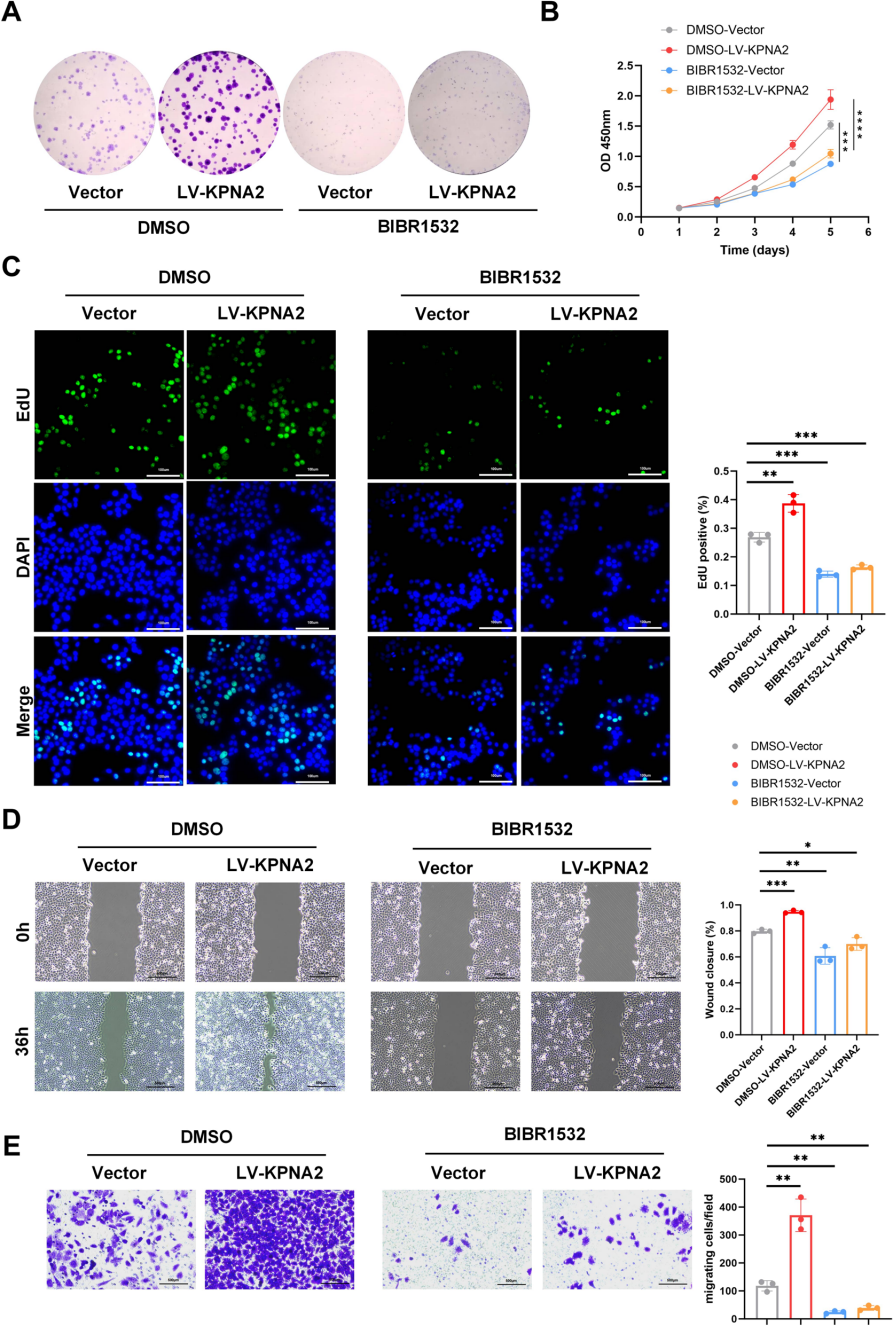
KPNA2 regulates HCC cell proliferation and metastasis through telomerase activation. Colony formation assays, **B** CCK8 assays, and **C** EdU assays indicated that telomerase inhibition could partly rescue the repressive effect caused by KPNA2 overexpression on cell proliferation in Hep3B cells. **D** Wound-healing assays and** E** transwell assays indicated that telomerase inhibition could partly rescue the repressive effect caused by KPNA2 overexpression on cell migration in Hep3B cells

Colony formation assays demonstrated that KPNA2 overexpression promoted cell proliferation, while telomerase inhibition partially abrogated KPNA2-induced proliferative effects. This finding was further supported by CCK-8 and EdU proliferation assays, which confirmed that inhibition of telomerase could partially reverse the proliferative advantage conferred by KPNA2 overexpression. To assess the effect of telomerase inhibition on cell migration, we performed wound healing and Transwell assays following treatment with BIBR1532 [26]. The results showed that both vector control and KPNA2-overexpressing HCC cells exhibited significantly reduced migration distances in the BIBR1532-treated group compared to the DMSO control. Consistently, the number of migrated cells in the Transwell assay was markedly lower in the BIBR1532 group than in the DMSO group. These findings suggest that telomerase inhibition partially counteracts the pro-migratory effects of KPNA2, indicating that KPNA2 may promote HCC cell migration through telomerase activation (see Fig. 6).

## Discussion

The activation of TMM is a vital feature of malignant tumors, especially in HCC. However, its prognostic impact and the underlying mechanisms remain unclear [6]. Therefore, comprehensive research and exploration of these mechanisms in HCC are necessary to identify novel biomarkers with prognostic impact and potential therapeutic value.

We concentrated on the TM-related genes. We firstly identified 224 differentially expressed TM-related genes from TCGA and ICGC databases, including 160 upregulated genes and 64 downregulated genes. We conducted further analysis via GO and KEGG functional enrichment analysis to show that these TM-related genes are significantly enriched in DNA replication, nuclear division, organelle fission, chromosomal region, catalytic activity, action on DNA, and cellular senescence. We identified those differentially expressed TM-related genes that play vital roles in the cell proliferation and cell cycle regulation. We further revealed eight hub TM-related genes, including *RNASEH2A*, *KPNA2*, *AURKB*, *FOXM1*, *MKI67*, *RAD54L*, *PLK1*, and *KIF4A*. Further analyses show that the expression level of hub TM-related genes is correlated with the key components of telomerase, the Shelterin complex, and genes implicated in the alternative lengthening of telomeres pathway.

Karyopherins are a group of proteins playing a central role in nuclear-cytoplasmic transport [27]. *KPNA2* belongs to the family of karyopherin alpha that is found in the protein network surrounding telomere repeat binding factors [28]. In the literature, the overexpression of *KPNA2* exists in many types of cancers, including HCC, associated with poor prognosis [29–31]. *KPNA2* was considered as a potential prognostic biomarker in HCC. However, the number of studies on the role of *KPNA2* in HCC is very limited. *AURKB* and *KIF4A* were found at the telomerase-positive cell line Hela by PICh, and *AURKB* was identified as a potential positive regulator of telomerase [32, 33]. *AURKB* is a widely expressed serine/threonine kinase. *AURKB* was overexpressed in various tumors and correlated with poor prognosis [34]. The selective inhibitor of *AURKB* was a promising molecular targeted therapy for HCC [35]. *KIF4A* is an N-type kinesin that functions in intracellular transport and cell division [36]. *KIF4A* was identified as an oncogene and associated with poor prognosis in multiple cancers. *RNASEH2A* and *FOXM1* were identified as TM-related genes since the yeast homologue's TM significance was validated. *FOXM1* promotes the progression of HCC by regulating the expression of *KIF4A*. Therefore, the *FOXM1-KIF4A* axis was considered a potential therapeutic target for HCC [37]. *MKI67*, *RAD54L*, and *PLK1* are potentially associated with telomeres and ALT pathways [38]. *MKI67* is also called *KI-67* antigen or *Ki-67*, which is a nuclear protein expressed only in G1, S, and G2-M phases of proliferating cells [39]. *Ki-67* has been widely investigated as a biomarker with prognostic value for many malignant diseases, including HCC [40]. *Ki-67* is expected as a therapeutic target for cancer therapy in the near future [41]. *RAD54L* plays an essential role in the DNA repair pathways. *RAD54L* germline mutations may increase the risk of developing cancer [42]. However, the mechanism of transcriptional regulation of *RAD54L* in HCC remains to be further studied. *PLK1* is essential for cellular growth and proliferation. *PLK1* inhibitors can cause cancer cell death by interfering with multiple phases of mitosis, which has been widely investigated in various cancer trials. In addition, there is evidence for a correlation between *PLK1* and cancer chemotherapy drug resistance, including paclitaxel and gemcitabine. It suggests that *PLK1* is an underlying therapeutic target for chemotherapy-resistant cancer [43]. Those results support the fact that the eight hub TM-related genes are critical in the development of HCC and can be used as valuable clinical biomarkers for the prognosis and therapeutics of HCC.

We also constructed and validated a simple model including seven TM-related genes. Among these seven genes, *KPNA2*, *CACNA1B*, *IRAK1*, and *CDCA8* were negative associated with survival, while *RGMA*, *ETS2*, and *GNE* were positively prognostic markers. We also provided a robust TM-related genes nomogram model, which showed better utility in supporting clinical decisions than tumor stage or other strategies. *ETS2* has been proven to exhibit both carcinogenic and suppressive effects in different types of carcinomas. In our research, *ETS2* is down-regulated in HCC, which is negative associated with survival. Besides, human telomerase promoter has new ETS sites after mutation [12], and *ETS2* was identified as telomerase activating and repressive factor [44]. *GNE* and *IRAK1* were identified as potential positive regulators of telomerase [33]. *GNE* is widely expressed in mammalian cells, with the highest expression level in tumor cells and liver cells [45]. *IRAK1* was overexpressed in HCC tumor tissues and may play a carcinogenic effect in HCC through the *TLR-IRAK* pathways [46]. The pharmaceutical *IRAK1* inhibitor was a promising novel strategy for HCC therapy [47]. *CACNA1B*, *CDCA8*, and *RGMA* were identified as prognostic factors and a promising therapeutical target in breast cancer [48–50]. However, except *IRAK1*, few studies on the expression and function of *ETS2*, *GNE*, *CACNA1B*, *CDCA8*, and *RGMA* in HCC have been reported.

Interestingly, *KPNA2* is a gene shared by eight hub TM-related genes and the 7-gene prognostic signature. We validated that *KPNA2* is involved in telomere maintenance in HCC cells and plays a significant role in regulating tumor proliferation and migration. These findings suggest that *KPNA2* functions as a critical mediator in both telomere biology and HCC progression, targeting the *KPNA2* nuclear transport pathway may inhibit tumor growth by disrupting the localization of key oncogenic proteins and telomerase components and may serve as a promising therapeutic target for liver cancer.

## Conclusions

In summary, this study identified eight TM-related hub genes with prognostic significance in HCC and established a novel TM-related gene signature. Furthermore, we validated *KPNA2* as a key regulator of telomere maintenance and tumor progression in HCC, suggesting it as a potential therapeutic target for improving clinical management of HCC.

## Supplementary Information

Additional file 1.

Additional file 2.

Additional file 3.

Additional file 4.

Additional file 5.

Additional file 6.

Additional file 7.

## Data Availability

No datasets were generated or analysed during the current study.
