# Supplementary material for: Bioinformatics analysis and experimental studies reveal KPNA2 as a novel biomarker of hepatocellular carcinoma progression and telomere maintenance
Source: Eur J Med Res. 2025 Jul 16;30:628. doi: 10.1186/s40001-025-02866-z (PMC12265345; doi:10.1186/s40001-025-02866-z)
Supplement: Supplementary file 1 — Additional file 1. [file 40001_2025_2866_MOESM1_ESM.docx]

**
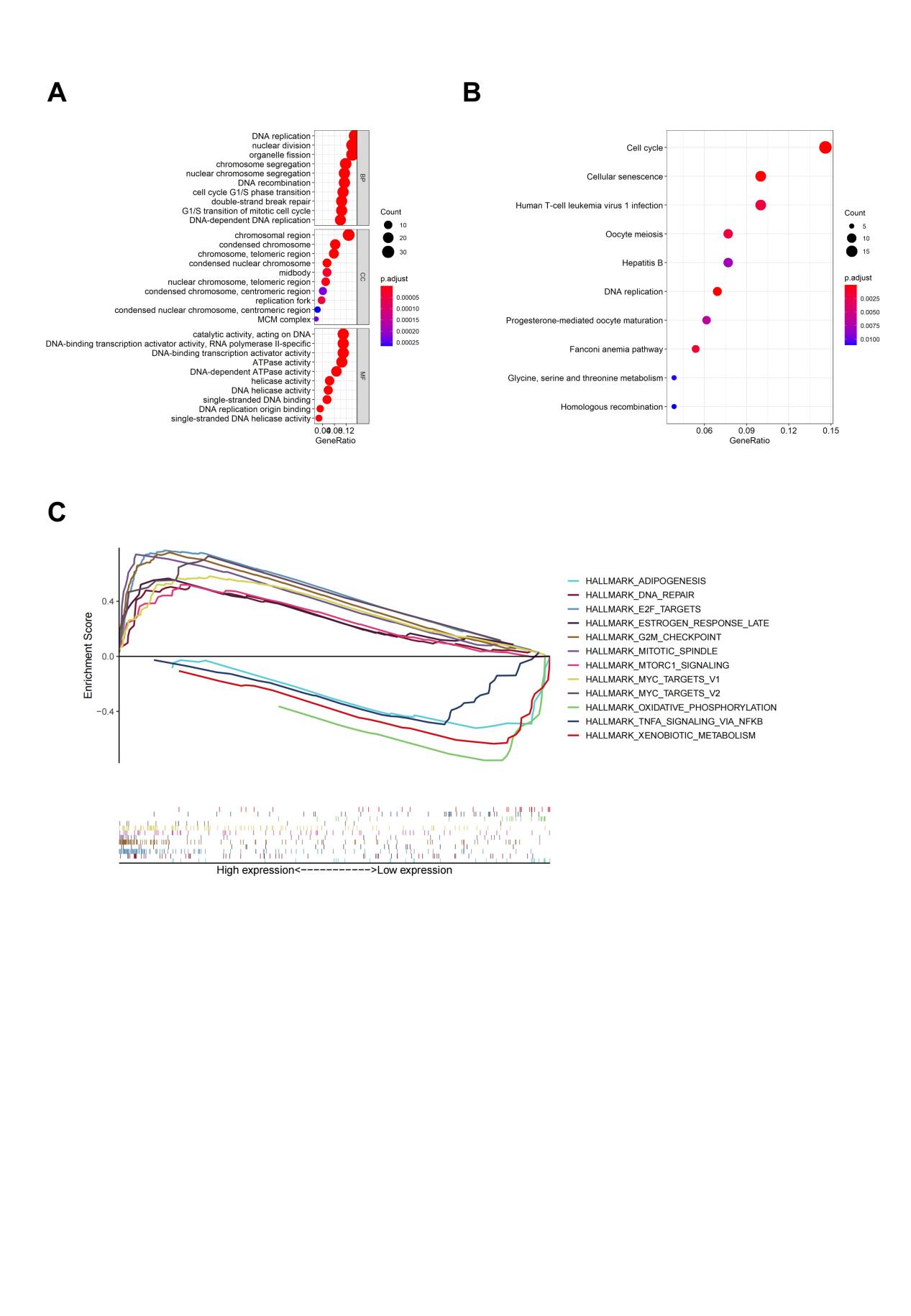
**

**Supplementary Figure 1.** Functional analysis of TM-related genes. **(A)** The top 10 most significant BP, CC, and MF terms in GO analysis. BP: biological process; CC: cellular component; MF: molecular function. **(B)** The top 10 most significant KEGG pathways. **(C)** GSEA analysis revealed significant enrichment in the cell cycle and cellular senescence pathways in the high-risk group.

**
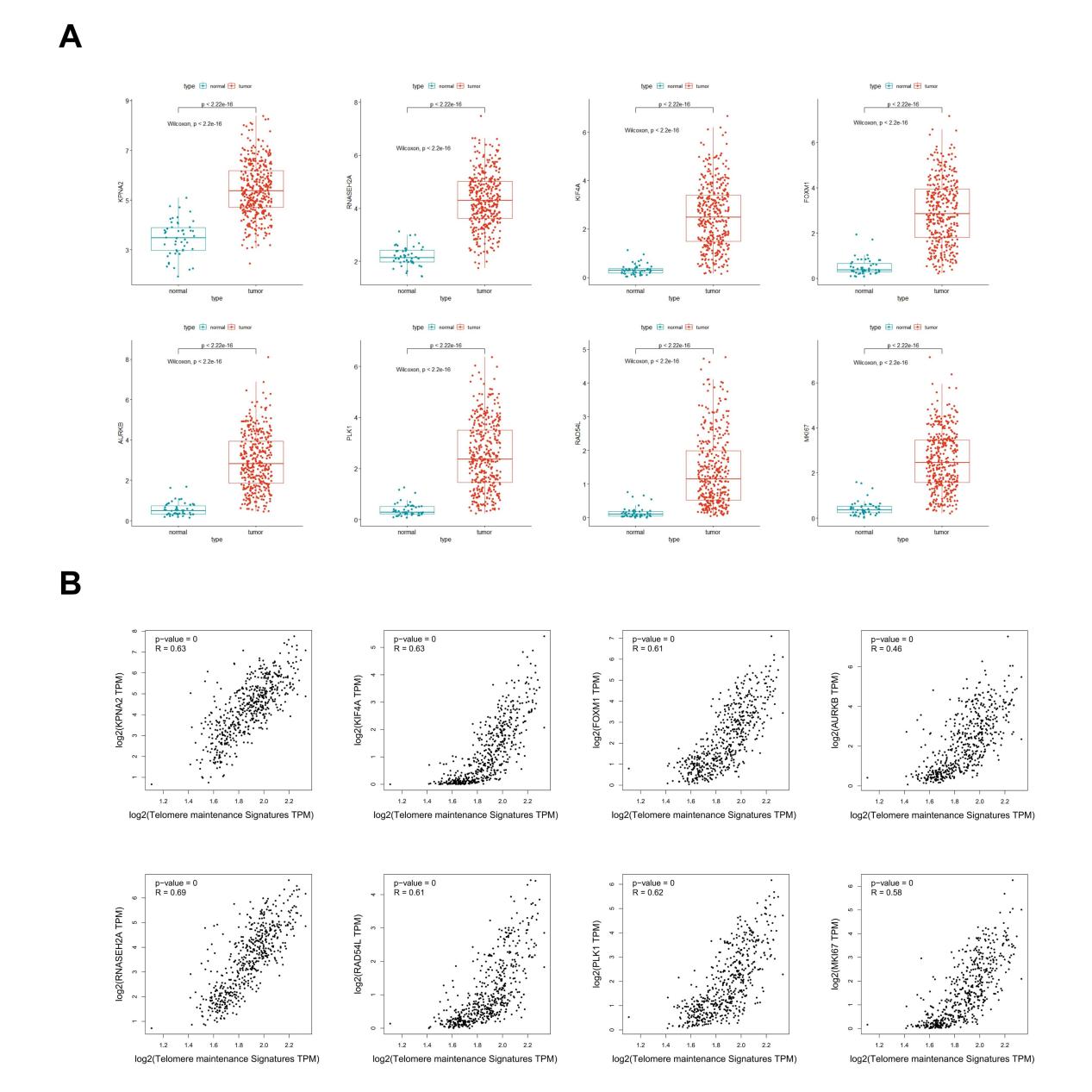
**

**Supplementary Figure 2.** Differential expression and correlation analysis of eight hub TM-related genes. **(A)** The mRNA expression levels of the eight genes in the TCGA database. **(B)** The relationship between eight genes and telomerase-related signature.


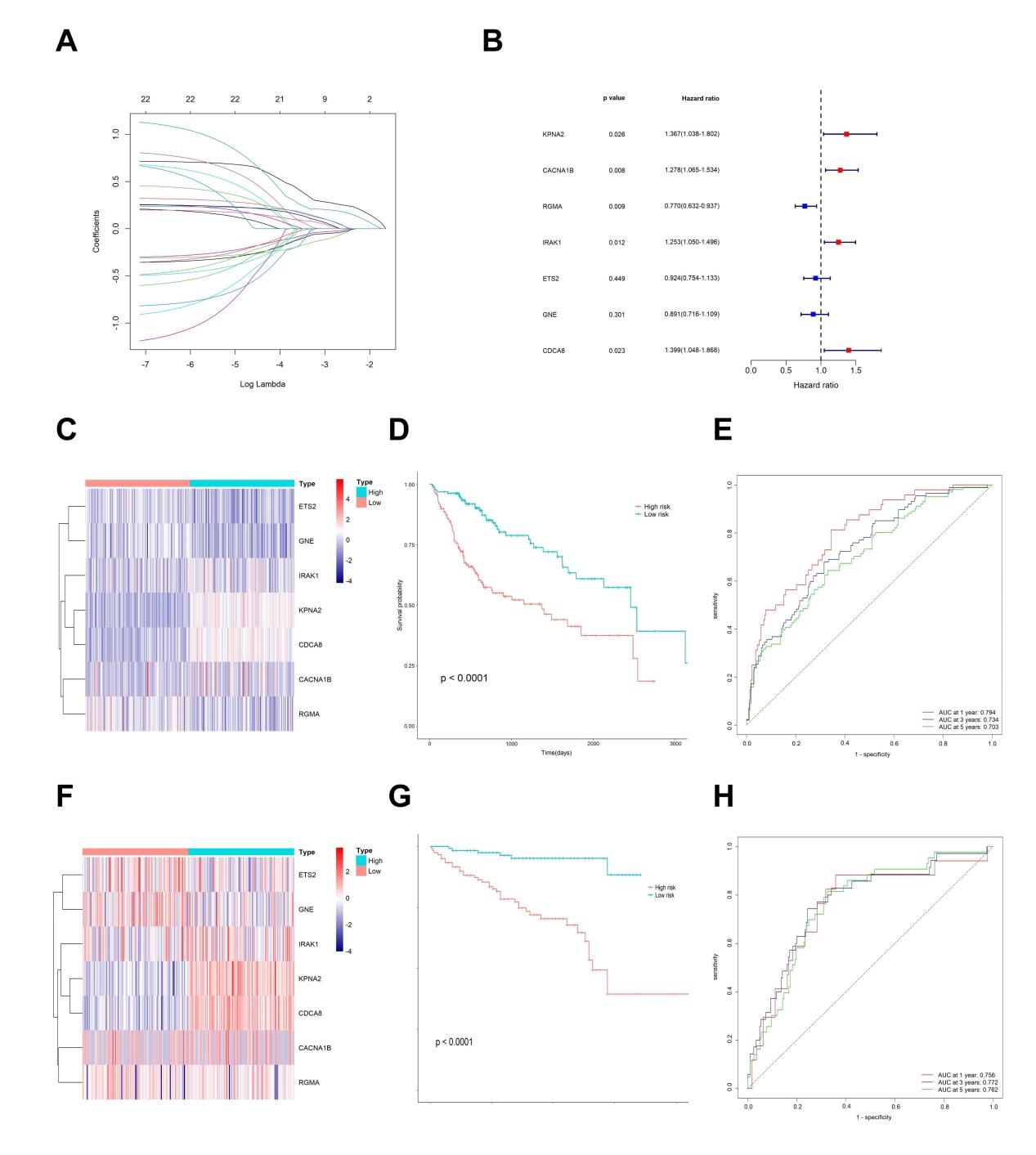


**Supplementary Figure 3.** Construction and validation of the prognostic TM-related gene signature. **(A)** The process of screening λ through 10-fold cross-validation in the LASSO model. **(B)** Univariate cox regression analyses of the 7 prognostic markers. **(C)** The expression patterns of the 7 prognostic markers within the TCGA cohort are illustrated in the heatmap. **(D)** Survival analysis of patients in the high-risk group and low-risk group in the TCGA cohort. **(E)** Time-dependent ROC curve verification of the prognostic signature at 1, 3, 5 years of overall survival (OS) in the TCGA cohort. **(F-H)** The heatmap, survival analysis and ROC curve of the prognostic signature in the ICGC cohort.


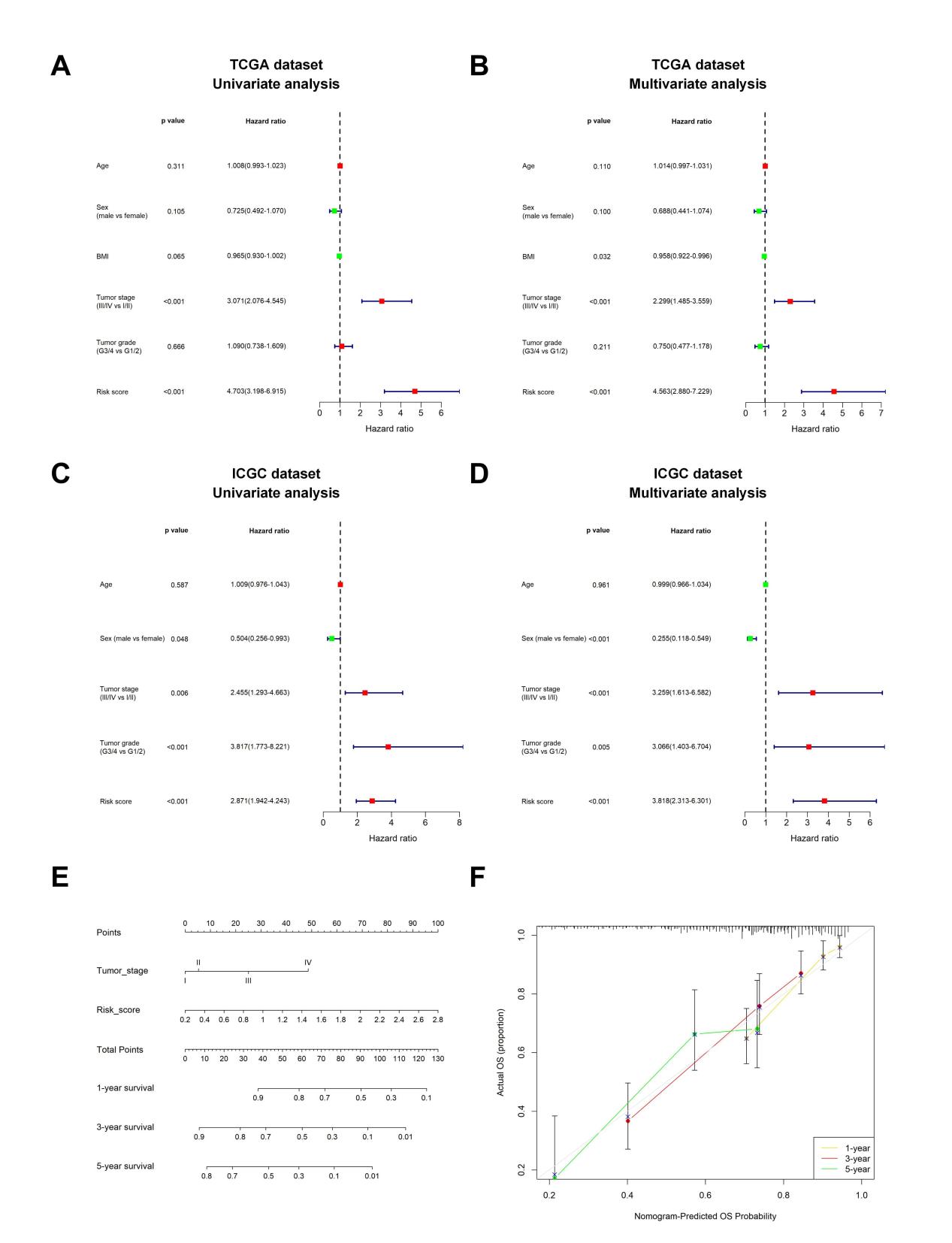


**Supplementary Figure 4.** Evaluating the independent role of the prognostic signature. **(A, B)** Univariate and multivariate cox regression analyses of the prognostic signature in the TCGA cohort. **(C, D)** Univariate and multivariate cox regression analyses of the prognostic signature in the ICGC cohort. **(E)** A nomogram to predict the survival probability at 1-, 3-, and 5-year. **(F)** The calibration plot for the nomogram predicting 1-, 3-, and 5-year overall survival.
