# Supplementary material for: Bioinformatics analysis and experimental studies reveal KPNA2 as a novel biomarker of hepatocellular carcinoma progression and telomere maintenance
Source: Eur J Med Res. 2025 Jul 16;30:628. doi: 10.1186/s40001-025-02866-z (PMC12265345; doi:10.1186/s40001-025-02866-z)
Supplement: Supplementary file 2 — Additional file 2. [file 40001_2025_2866_MOESM2_ESM.docx]

**Supplementary Table 1.** shRNA sequences for KPNA2 knockdown.

| Name | Sequences |
| --- | --- |
| Homo-KPNA2-sh1 | GCTGGTTTGATTCCGAAATTT |
| Homo-KPNA2-sh2 | CTACCTCTGAAGGCTACACTT |
| Homo-KPNA2-sh3 | ACATTGTCAAAGGCATAAATA |
