# Supplementary material for: Bioinformatics analysis and experimental studies reveal KPNA2 as a novel biomarker of hepatocellular carcinoma progression and telomere maintenance
Source: Eur J Med Res. 2025 Jul 16;30:628. doi: 10.1186/s40001-025-02866-z (PMC12265345; doi:10.1186/s40001-025-02866-z)
Supplement: Supplementary file 3 — Additional file 3. [file 40001_2025_2866_MOESM3_ESM.docx]

**Supplementary Table 2.** Summary of primer sequence for qRT-PCR.

| Gene name |  | Sequence |
| --- | --- | --- |
| KPNA2 | Forward Primer | CTGCCCGTCTTCACAGATTCA |
|  | Reverse Primer | GCGGAGAAGTAGCATCATCAGG |
| TERT | Forward Primer | AAATGCGGCCCCTGTTTCT |
|  | Reverse Primer | CAGTGCGTCTTGAGGAGCA |
| TERF1 | Forward Primer | TGCCGACCCTACTGAGGAG |
|  | Reverse Primer | GCAGAGGAAATCGAGCATCCA |
| TERF2 | Forward Primer | AGAGGCAGTCAATCGCTGG |
|  | Reverse Primer | GAAGTCCCCGTACCGGCTA |
