# Supplementary material for: Bioinformatics analysis and experimental studies reveal KPNA2 as a novel biomarker of hepatocellular carcinoma progression and telomere maintenance
Source: Eur J Med Res. 2025 Jul 16;30:628. doi: 10.1186/s40001-025-02866-z (PMC12265345; doi:10.1186/s40001-025-02866-z)
Supplement: Supplementary file 4 — Additional file 4. [file 40001_2025_2866_MOESM4_ESM.docx]

**Supplementary Table 3.** Antibody information.

| Name | Cat No. |
| --- | --- |
| KPNA2 Monoclonal antibody | 66870-1-Ig, Proteintech, China |
| TERT Recombinant antibody | 84636-1-RR, Proteintech, China |
| GAPDH Monoclonal antibody | 60004-1-Ig, Proteintech, China |
