# Supplementary material for: Bioinformatics analysis and experimental studies reveal KPNA2 as a novel biomarker of hepatocellular carcinoma progression and telomere maintenance
Source: Eur J Med Res. 2025 Jul 16;30:628. doi: 10.1186/s40001-025-02866-z (PMC12265345; doi:10.1186/s40001-025-02866-z)
Supplement: Supplementary file 5 — Additional file 5. [file 40001_2025_2866_MOESM5_ESM.docx]

**Table1.** Baseline characteristics in the TCGA-LIHC cohort and ICGC (LIRI-JP) cohort.

|  | TCGA cohort | LIRI-JP cohort |
| --- | --- | --- |
| No. of patients | 318 | 240 |
| Age (Median/ Range) | 61/16-82 | 69/31-89 |
| Sex |  |  |
| Male | 219 | 179 |
| Female | 99 | 61 |
| Prior malignancy |  |  |
| Yes | 28 | 32 |
| No | 290 | 208 |
| Tumor stage |  |  |
| Stage I | 160 | 36 |
| Stage II | 76 | 109 |
| Stage III | 79 | 74 |
| Stage IV | 3 | 21 |
| Tumor grade |  |  |
| G1 | 43 | 31 |
| G2 | 153 | 165 |
| G3 | 108 | 23 |
| G4 | 12 | 1 |
| Not reported | 2 | 20 |
| Survival status |  |  |
| Alive | 209 | 197 |
| Dead | 109 | 43 |
| OS (median days) | 605 | 780 |
