# Supplementary material for: Bioinformatics analysis and experimental studies reveal KPNA2 as a novel biomarker of hepatocellular carcinoma progression and telomere maintenance
Source: Eur J Med Res. 2025 Jul 16;30:628. doi: 10.1186/s40001-025-02866-z (PMC12265345; doi:10.1186/s40001-025-02866-z)
Supplement: Supplementary file 6 — Additional file 6. [file 40001_2025_2866_MOESM6_ESM.docx]

**Supplementary Table 4.** Genes associated with the prognosis of patients by univariate Cox regression analysis.

|  | HR | HR.95L | HR.95H | pvalue |
| --- | --- | --- | --- | --- |
| E2F2 | 1.523387 | 1.249161 | 1.857813 | 3.22E-05 |
| MYOM2 | 0.705976 | 0.580124 | 0.859132 | 0.00051 |
| RAD51 | 1.416968 | 1.170217 | 1.71575 | 0.000357 |
| KLF6 | 0.770139 | 0.63182 | 0.938738 | 0.009714 |
| TRIP13 | 1.760761 | 1.44799 | 2.141091 | 1.43E-08 |
| HMMR | 1.584692 | 1.294921 | 1.939308 | 7.88E-06 |
| MCM2 | 1.589519 | 1.297946 | 1.946592 | 7.39E-06 |
| MCM6 | 1.551636 | 1.277939 | 1.883951 | 9.13E-06 |
| PAFAH1B3 | 1.398663 | 1.158373 | 1.688799 | 0.000486 |
| PGM1 | 0.790799 | 0.668189 | 0.935909 | 0.006323 |
| NDC80 | 1.735798 | 1.398158 | 2.154973 | 5.83E-07 |
| ORC1 | 1.560431 | 1.283626 | 1.896925 | 7.97E-06 |
| RAD54L | 1.585715 | 1.302059 | 1.931166 | 4.54E-06 |
| MT3 | 1.304331 | 1.096655 | 1.551334 | 0.002676 |
| AURKA | 1.427473 | 1.186828 | 1.716912 | 0.000158 |
| KIF4A | 1.695742 | 1.369228 | 2.100119 | 1.30E-06 |
| ORC6 | 1.527115 | 1.259664 | 1.851351 | 1.63E-05 |
| ESR1 | 0.631552 | 0.518124 | 0.769812 | 5.36E-06 |
| CLSPN | 1.569782 | 1.29385 | 1.904561 | 4.83E-06 |
| CDC45 | 1.526806 | 1.256988 | 1.854543 | 2.00E-05 |
| MSH2 | 1.362051 | 1.101765 | 1.683828 | 0.004295 |
| GSTZ1 | 0.726309 | 0.598565 | 0.881316 | 0.001195 |
| E2F1 | 1.394568 | 1.158339 | 1.678972 | 0.000444 |
| MCM4 | 1.40067 | 1.159891 | 1.691432 | 0.000463 |
| RNASEH2A | 1.349801 | 1.120578 | 1.625913 | 0.001584 |
| ASF1B | 1.432816 | 1.173334 | 1.749681 | 0.000419 |
| EZH2 | 1.743056 | 1.417942 | 2.142714 | 1.32E-07 |
| NCAPG | 1.743664 | 1.400423 | 2.171034 | 6.66E-07 |
| FOXM1 | 1.586585 | 1.29142 | 1.949213 | 1.11E-05 |
| RAD51AP1 | 1.402002 | 1.151307 | 1.707285 | 0.000775 |
| ALDH2 | 0.628829 | 0.518361 | 0.76284 | 2.52E-06 |
| FANCE | 1.415725 | 1.170651 | 1.712105 | 0.000338 |
| MCM3 | 1.364311 | 1.126773 | 1.651924 | 0.001458 |
| HRG | 0.799237 | 0.677325 | 0.943092 | 0.007959 |
| ECT2 | 1.52082 | 1.252771 | 1.846222 | 2.26E-05 |
| DNAJC6 | 1.289852 | 1.066043 | 1.560649 | 0.008853 |
| CDC20 | 1.779654 | 1.452007 | 2.181234 | 2.82E-08 |
| NEK2 | 1.536041 | 1.246037 | 1.89354 | 5.81E-05 |
| CENPF | 1.494239 | 1.224063 | 1.824048 | 7.92E-05 |
| ELOVL3 | 1.410271 | 1.176764 | 1.690114 | 0.000197 |
| EPHX2 | 0.782735 | 0.662992 | 0.924104 | 0.003831 |
| PCK1 | 0.73284 | 0.613265 | 0.875731 | 0.000626 |
| GNMT | 0.750172 | 0.624283 | 0.901447 | 0.002162 |
| FOSB | 0.746905 | 0.61007 | 0.914432 | 0.004708 |
| CTAG2 | 1.322463 | 1.103513 | 1.584855 | 0.002473 |
| PKMYT1 | 1.386746 | 1.157377 | 1.661571 | 0.000394 |
| ASS1 | 0.765466 | 0.630156 | 0.92983 | 0.00708 |
| PNCK | 1.531276 | 1.285569 | 1.823945 | 1.80E-06 |
| SYNE1 | 0.741116 | 0.628216 | 0.874306 | 0.000381 |
| TOP2A | 1.543407 | 1.257616 | 1.894144 | 3.27E-05 |
| CCNB1 | 1.784082 | 1.440078 | 2.210261 | 1.18E-07 |
| CDCA8 | 1.888685 | 1.521997 | 2.343716 | 7.75E-09 |
| DSCC1 | 1.295061 | 1.076783 | 1.557586 | 0.006042 |
| HMGA1 | 1.439258 | 1.198879 | 1.727834 | 9.41E-05 |
| SORL1 | 0.776672 | 0.658458 | 0.916108 | 0.002699 |
| PIF1 | 1.449095 | 1.184829 | 1.772303 | 0.000305 |
| FANCD2 | 1.417769 | 1.175809 | 1.709519 | 0.000256 |
| CCNA2 | 1.495605 | 1.259713 | 1.775669 | 4.30E-06 |
| PSPH | 1.40605 | 1.151438 | 1.716965 | 0.000828 |
| MAGEA4 | 1.312393 | 1.130053 | 1.524154 | 0.000368 |
| CACNA1B | 1.293158 | 1.093878 | 1.528742 | 0.002606 |
| MKI67 | 1.690432 | 1.37067 | 2.084791 | 9.24E-07 |
| INCENP | 1.332719 | 1.091034 | 1.627942 | 0.004902 |
| CHEK1 | 1.561324 | 1.272304 | 1.915998 | 1.99E-05 |
| FOXO1 | 0.626786 | 0.507056 | 0.774788 | 1.57E-05 |
| SLC7A11 | 1.289497 | 1.064317 | 1.562318 | 0.009417 |
| ADK | 0.749834 | 0.623968 | 0.90109 | 0.002134 |
| ETS2 | 0.715344 | 0.599011 | 0.854269 | 0.000216 |
| CHAF1B | 1.460826 | 1.203763 | 1.772786 | 0.000124 |
| GNE | 0.732584 | 0.621152 | 0.864007 | 0.000219 |
| AZGP1 | 0.804707 | 0.688982 | 0.939869 | 0.006092 |
| RECQL4 | 1.347079 | 1.144303 | 1.585787 | 0.000344 |
| RACGAP1 | 1.536505 | 1.263698 | 1.868205 | 1.66E-05 |
| AKR7A3 | 0.744465 | 0.625977 | 0.885382 | 0.000849 |
| ALPL | 0.715261 | 0.593708 | 0.8617 | 0.000421 |
| TDRD5 | 1.333309 | 1.12781 | 1.576253 | 0.000756 |
| HAAO | 0.773287 | 0.645387 | 0.926533 | 0.005317 |
| CCT3 | 1.246945 | 1.058909 | 1.468372 | 0.008138 |
| RFC4 | 1.413631 | 1.177596 | 1.696975 | 0.000204 |
| TKT | 1.295009 | 1.076683 | 1.557607 | 0.006064 |
| CDC25A | 1.524774 | 1.263597 | 1.839933 | 1.08E-05 |
| PLK1 | 1.819006 | 1.465872 | 2.257213 | 5.55E-08 |
| DTYMK | 1.41167 | 1.203298 | 1.656127 | 2.33E-05 |
| FEN1 | 1.417602 | 1.187907 | 1.691711 | 0.000109 |
| AR | 0.711267 | 0.600359 | 0.842663 | 8.17E-05 |
| UGP2 | 0.67361 | 0.548617 | 0.827079 | 0.000161 |
| CDK1 | 1.67064 | 1.356239 | 2.057924 | 1.40E-06 |
| EXO1 | 1.611346 | 1.297359 | 2.001324 | 1.60E-05 |
| BRSK2 | 1.288628 | 1.069224 | 1.553053 | 0.007749 |
| RMI2 | 1.28478 | 1.083987 | 1.522768 | 0.003852 |
| ETV4 | 1.353752 | 1.11515 | 1.643407 | 0.002201 |
| SHMT1 | 0.774147 | 0.65251 | 0.918459 | 0.003333 |
| AURKB | 1.466292 | 1.213839 | 1.771251 | 7.18E-05 |
| TPRXL | 1.325853 | 1.120929 | 1.568241 | 0.000993 |
| FANCB | 1.464815 | 1.202531 | 1.784305 | 0.000149 |
| RGMA | 0.722094 | 0.60351 | 0.863978 | 0.000375 |
| KPNA2 | 1.905871 | 1.55759 | 2.332029 | 3.75E-10 |
| IRAK1 | 1.302631 | 1.091059 | 1.55523 | 0.003459 |
| XRCC2 | 1.456605 | 1.187798 | 1.786244 | 0.000302 |
| PRIM1 | 1.292085 | 1.063936 | 1.569156 | 0.009732 |
| WDHD1 | 1.498016 | 1.230279 | 1.82402 | 5.75E-05 |
| PAGE2 | 1.327276 | 1.115209 | 1.579669 | 0.001434 |
