# Supplementary material for: Bioinformatics analysis and experimental studies reveal KPNA2 as a novel biomarker of hepatocellular carcinoma progression and telomere maintenance
Source: Eur J Med Res. 2025 Jul 16;30:628. doi: 10.1186/s40001-025-02866-z (PMC12265345; doi:10.1186/s40001-025-02866-z)
Supplement: Supplementary file 7 — Additional file 7. [file 40001_2025_2866_MOESM7_ESM.docx]

**Table2.** The Identification of a prognostic signature based on TM-related genes in the TCGA cohort.

|  | Univariate Cox regression analysis | | | | LASSO coefficient |
| --- | --- | --- | --- | --- | --- |
|  | HR | HR.95L | HR.95H | pvalue |  |
| CDCA8 | 1.888684593 | 1.521997252 | 2.343716119 | 7.75E-09 | 0.168401787 |
| CACNA1B | 1.293157995 | 1.093878167 | 1.528742095 | 0.002605579 | 0.009296239 |
| ETS2 | 0.7153439 | 0.599011233 | 0.854269281 | 0.000216152 | -0.007062245 |
| GNE | 0.732583895 | 0.621151649 | 0.864006663 | 0.000218778 | -0.023405336 |
| RGMA | 0.722093513 | 0.603509537 | 0.86397813 | 0.000374595 | -0.031361508 |
| KPNA2 | 1.905871056 | 1.557590024 | 2.332028599 | 3.75E-10 | 0.243777819 |
| IRAK1 | 1.302630962 | 1.091058884 | 1.555229924 | 0.00345909 | 0.012413379 |
